# Increased risk of cancer among relatives of patients with lung cancer in China

**DOI:** 10.1186/1471-2407-5-146

**Published:** 2005-11-11

**Authors:** Yongtang Jin, Yingchun Xu, Ming Xu, Saoli Xue

**Affiliations:** 1 School of Public Health, Anhui Medical University, Hefei 230032, Anhui province, P R China; 2 Department of Pharmacology, Anhui Medical University, Hefei 230032, P R China; 3 Department of Biotechnology, Anhui Medical University, Hefei 230032, P R China

## Abstract

**Background:**

Genetic factors were considered as one of the risk factors for lung cancer or other cancers. The aim of this work was to determine whether a genetic predisposition accounts for such familial aggregation of cancer among relatives of lung cancer probands.

**Methods:**

A case-control study was conducted in 800 case families identified by lung cancer patients (probands), and in 800 control families identified by the probands'spouses. The data were analysed with logistic regression analysis model.

**Results:**

The data revealed a significantly greater overall risk of cancer (OR = 1.82, P < 0.01) in the proband group. The relatives of lung cancer probands maintained an increased risk of non-lung cancer (P < 0.05) after adjusting for confounder factors. The crude odds ratio of a proband family having one family member with cancer was 1.67 compared with control families. Proband families were 2.56 times more likely to have two other family members with cancer. For three cancers and four or more cancers, the risk increased to 3.50 and 5.91, respectively. The most striking differences in cancer prevalence between proband and control families were noted for cancer risk among female relatives. The strongest effects were for not only lung cancer in any female relatives (OR 2.17, 95%CI 1.60–3.64) and mothers (OR 2.78, 95%CI 1.23–5.12) and sisters (OR 2.03, 95%CI 1.26–3.97), but also non-lung cancer in females and mothers (OR 2.00, 95%CI 1.26–3.01, and OR 2.34, 95%CI 1.28–4.40, respectively).

**Conclusion:**

These data support the hypothesis of a genetic susceptibility to cancer in families with lung cancer, and the female genetic susceptibility to cancer might be greater than male.

## Background

Inasmuch as some 22 per cent of all persons die of cancers affecting various anatomic sites, the random occurrence of cancer among several members of a nuclear family would be usual [1]. However, when cancers occur among members in several generations of the same family, chance itself may not be sufficient to account for that occurrence. An increased familial risk for cancer and particularly lung cancer has been demonstrated among relatives of lung cancer patients [2-6]. However, the precise genetic characteristics which influence lung cancer susceptibility are not known. Many studies of families may be used to attempt to disentangle the effects of shared environmental risk factors and genetic factors [7-9].

However, these conclusions do not address the occasionally observed increased frequency of cancers of several sites in combination, such as those of the breast, brain, bone, liver, adrenal cortex, blood, and/or lymphatics[2]. Warthin[10] was the first to describe a large kindred having a remarkable high incidence of cancers at various sites. Pedigree analysis of several large families with multiple cancers across several generations led Lynch and Krush[11] to propose a new genetic entity, later called "cancer family syndrome". In general, those studies showed that the familial occurrence of cancer tends to deviate from the Possion distribution, in that more families than expected by chance have two or more affected, closely related members. However, these high-risk families were selected for study because of their high frequency of cancer cases. Because the laws of probability can not preclude the probability that these cancer families merely represent a chance occurrence, some investigators have questioned genetic susceptibility as a basis for familial aggregation of cancers at various sites.

To explore whether familial aggregation of cancer represents a random or specific event, we conducted a retrospective case-control study of families. The lung cancer patient (proband) was used to identify a case family and the proband's spouse was used to identify a control family in rural regions of Anhui, China.

## Methods

### Study population

Case-probands were selected if they satisfied a defined eligibility criteria, namely: all farmers who had died of a primary malignancy of the lung (stated as an underlying cause of death in their death records) in three counties in Anhui over a ten-year period (1995–2004). Spouses of probands were included in the control group if these spouses were themselves unaffected with lung cancer and were farmers. The families of probands and controls have lived in rural regions for over 20-year.

The population of the rural regions in Anhui had almost little migration 20 years ago and the cultural patterns and ethnicity have retained definite demographic characteristics. The use of spouse-matched controls can be expected to control for the potential confouning effects of different cultural and residential environment, e.g. diet, drinking water, socioeconomic status and so on.

First-degree relatives (parents, full siblings) of probands were designated as case families. The control families comprised first-degree relatives (parents, full siblings) of the spouses of probands. Thus, in the following sections of this paper, the term "family", when applied to the study population, never includes the proband or spouse.

### Data collection

A complete listing of all deaths satisfying the eligibility criteria was obtained from the local Tumor Prevention and Treatment Office of Health Bureau in rural area. Standard demographic characteristics of the probands and the identities of their next-of-kin were obtained from the death records of probands. Local community doctors were recruited to help initiate contact with family members of the case-proband. Provided with the identity of the proband's next-of-kin as well as the usual address of the deceased, the doctors generated a list of addresses of all family members in each pedigree.

Trained interviewers with standard protocols obtained information on each member of the family by face-to-face interviews from (in order of preference) the involved persons (except the proband or other deceased family member), spouse, parent, sibling, or adult offspring. Cancer histories were verified by two methods: 1) a review of death certificates on a sample (90.4 and 88.4 percent, respectively) of relatives of probands and spouses who were reported to have died in rural regions of Anhui and 2) corroborative information from additional family contacts. Because only first-degree relatives (parent and siblings) were included in the study, bias introduced by inability to verify all cancer deaths should be minimal[12].

For the protection of human subjects, all of the subjects in this study signed a consent form according to the guidelines of the World Medical Association Declaration of Helsinki.

### Data analysis

Frequency statistics of the study population were computed. Mean age differences between proband and spouse relatives were tested for signficance using two-sided t tests. To determine whether the distribution of relatives was equivalent between study groups, contingency table chisquare tests were used. for each family, design variables were assigned as 1 for the presence and 0 for the absence of each of the following among the proband's or spouse's relatives: one, two, three, four or more cancers. Logistic regression analysis was used to the data to predict whether a family belonged to the case or control group based on these design variables. The resulting regression coefficients (β_i_)were used to calculate the relative risks of cancer by the formula: odd ratio = e^βi^, for the ith variables. To determine whether differences in environmental exposure between the case an control families could explain the difference in cancer occurrence, another logistic model was fitted to predict cancer occurrence in each family member based on age, sex, the occupation/industrial exposure pack-years of tobacco exposure and a variable that expressed family membership (case or control). Because the excess risk of cancer in the proband families remained statistically significant, a test of homogeneity was performed to determine whether the study groups differed in their distribution of cancer types. To isolate where these differences occurred, contingency chisquare tests were applied.

Crude odds ratios (OR) were calculated as estimates of the relative risks, and Woolf's method was used to determine 95% confidence intervals (CI)[13]. Maximum likelihood estimates of adjusted ORs were obtained from unconditional logistic regression analysis by the PHREG procedure in SAS software[14].

These variables examined as possible confounders and effect modifiers included: number of first-degree relatives (2–4, 5–7, 8–10, >10); smoking status (never, ex-smoker, current smoker); smoking duration (never, 1–29 yr, 30–45 yr, >45 yr); amount smoked (none, <20 cigarettes per day (cpd), 20–30 cpd, 31–40 cpd, >40 cpd); pack-years (defined as cigarette packs smoked daily multiplied by years of smoking, gram equivalents of leaf tobacco. Assuming 1 g per cigarette) of smoking (none, >0–20, >20–40, >40 pack-years); residence (females only) (selected three counties vs other counties); age (<55 yr, 55–65 yr, >65 yr); ethnicity (Han vs others); sex, passive smoking exposure (ever/never and by total years); education (through high school vs greater than high school and by level: none, primary school, middle school, high school, >high school); martital status (never married, married, and widowed, divorced or separated); high-risk industry/occupation (employed in jobs with exposure to asbestos, benzene, beryllium, bischloromethyl ether, ceramic dust, talc, chemical fertilizers, chromium or chromates, coke oven emissions, dyes, glues, lacquers, fiberglass, cotton dust, insecticides, pesticides, herbicides, fungicides, isopropyl oil, paint sprays, petroleum products, radioactive materials, vinylchloride or explosives); the cumulative exposure to smoky coal use for a given individual was determined by multiplying the annual rate of smoky coal use times the number of years (coal consumption was generally fixed for the households over the life cycle of the family and three exposure categories were formed: >0–70, 70–140, and >140 tons); alcohol consumption (ever/never); vital status; and type of respondent (self/spouse/other); history of COPD (yes/no).

## Results

In our study, 800 eligible probands were identified. These families were only included in the data set once (if there are more than one cases in a family, the earliest case diagnosed was selected as the proband). An average of 2.6 and 2.9 interviews (contacts) were made to complete information on each of these proband and spouse families, respectively. The largest proportion of the contacts was through siblings, followed closely by spouses. Less than 17 per cent of the contacts were from adult offspring and surviving parents. The distributions of reported cancer in relatives by source of contacts were not significantly different between proband and spouse families. Information on complete two-generation pedigrees was obtained for 800 case families and 800 control families. The remaining families of probands and spouses were excluded from the analysis because of: inadequate information in names, addresses, incomplete data on each individual in the pedigree (2.8 per cent), or refusals to participate (4.2 per cent). The resulting sample includes 4,453 case family members and 4,378 control family members.

A total of 390 case family members(8.76 per cent) and 212 control family members (4.84 per cent) were reported to have cancer (only primary malignancy counted). 36(9.2 per cent) and 22 (10.4 per cent) were living cancer cases in the case and control groups, respectively. Of the cancer deaths reported in the death certificates, 92.8 per cent were detected by interview. Analogously, 94.2 per cent of the noncancer deaths were reported. There were no significant differences in age at death or age of living family members with cancer between the two study groups.

Male probands almost numbered equally female probands by a ratio of 1.12 to 1.05. The mean family size was similar for both groups, 5.6 including the proband, and 5.5 including the spouse, respectively. Although more of the probands were male and more were older than their spouses, no significant sex or age differences were observed between the two groups of relatives (table 1). The proportional distribution of types of relatives, however, was also non-different between the comparison groups.

**Table 1:** Characteristics of probands' and spouses' families

Family members and characteristic	Proband No. (%)	Mean age, yr, of proband	Spouse No. (%)	Mean age, yr, of spouse
Families	800		800	
All relatives	4,453		4,378	
Male relatives	2,484(100.0)		2,418(100.0)	
Living	1,043(42.0)	56.0	1,064(44.0)	55.3
Dead	1,441(58.0)	49.9	1,354(56.0)	59.4
Female relatives	1,969(100.0)		1,960(100.0)	
Living	650(33.0)	57.7	784(40.0)	60.0
Dead	1,319(67.0)	51.0	1,176(60.0)	57.9
Parents	1600(100.0)		1600(100.0)	
Living^a^	128(8.0)	66.8	192(12.0)	69.0
Dead	1,472(92.0)	60.5	1,408(88.0)	63.1
Siblings	2,853(100.0)		2,778(100.0)	
Living	1,883(66.0)	49.0	1,972(71.0)	50.8
Dead	970(34.0)	46.2	806(29.0)	49.0

^a ^P < 0.01, between proportion of relatives in proband and spouse groups who were parents.

Because the proband families tended to be slightly larger than spouse families, adjustment was made for the total number of relatives in the family when determining the familial risk of cancer. Case families were significantly more likely to contain at least one family member with any type of cancer (OR = 1.82, P < 0.01). Each family was further classified according to the total number of cancers present among its menbers (none, one, two, three, four or more). Proband families were 1.67 times more likely to have one family member with cancer and 2.56 times more likely to have two family members with cancer than spouse families(table 2). For three cancers and four or more cancers, the relative risk increased to 3.50 and 5.91, respectively. Each risk other than the lastest estimate was significant at the 0.01 level. The average age of those living family members with cancer, or deceased from cancer, is also presented. The differences are minimal.

**Table 2:** Familial risk of developing cancer (all sites) among lung cancer case families and control families

	Case			Control			
			
Cancers (no.)	Families (no.)	Relatives^a ^(no.)	Age^b ^(M)	Families (no.)	Relatives^a ^(no.)	Age^b ^(M)	OR^c ^(95% CI)
0	518	2369		627	2598		
1	209	902	62.2	143	814	61.8	1.67** (1.22–2.01)
2	51	251	61.0	22	112	60.2	2.56** (1.44–4.29)
3	21	95	62.2	7	37	63.1	3.50** (1.36–10.02)
4+	4	36	62.4	1	17	63.2	5.91 (0.79–132.10)

** p < 0.01

^a ^Excludes probands and spouses.

^b ^Mean age of members living with or deceased from cancer.

^c ^Represents familial risk of case families, not individual risk, after adjusting for family size, total smoky coal exposure, number of person smoking and commune of residence.

To determine whether this excess risk was evident for cancers, we tabulated the total number of cancers in a family including lung cancer (International Classification of Diseases (ICD), 9^th ^Revision, code nos. 162.0–162.9) (table 3). After adjusting for confounder factors, the overall relative risk of one member with cancer in case families (OR 1.54) remained statistically significant. The odds ratios for separate categories also remained significant for families with three members with cancer. There was a trend of risk increased for families with more and more members with cancer. In addition, table 3 significantly shows the increased risk of lung cancer among relatives of lung cancer patients(OR 1.4–5.6, P < 0.05), and the more number of relatives affected by lung cancer, the greater risk of lung cancer among the other relatives of lung cancer patients.

**Table 3:** Familial risk for developing cancer (non-lung cancer and lung cancer)among lung cancer case families and control families

	Case		Control		
			
Cancers (n.)	Families (no.)	Relatives^a ^(no.)	Families (no.)	Relatives^a ^(no.)	OR^b ^(95%CI)
Non-lung cancer					
0	657	3,591	720	3,865	
1	96	568	63	397	1.54(1.17–2.28)**
2	34	194	12	79	1.67 (0.91–3.91)
3	11	83	4	28	2.11 (1.02–7.98)*
4+	2	17	1	9	3.13 (0.89–60.66)
Lung cancer					
0	658	3629	706	3757	
1	113	636	81	528	1.48 (1.11–2.02)*
2	17	103	11	77	2.96 (1.44–6.02)**
3	10	69	2	16	5.67 (1.24–34.89)*
4+	2	16	0	0	--

* p < 0.05. ** p < 0.01.

^a ^Excludes probands and spouses. ^b ^Represents familial risk of case families, not individual risk, adjusted for family size, total smoky coal exposure, number of person smoking and commune of residence.

The results for the analyses that treated lung cancer, non-lung cancer and any cancer among relatives of probands as outcome variables are shown in table 4. Among the 4453 first-degree relatives of the probands, 185 (4.2%) had a reported diagnosis of lung cancer, and 205 (4.6%) had a diagnosis of a non-lung cancer. There were 109 (2.5%) lung cancer cases and 103 (2.4%) non-lung cancer cases among the 4378 first-degree relatives of the spouses. The strongest effects were for not only lung cancer in any female relatives (adjusted OR 2.17, 95%CI 1.60–3.64) and mothers (adjusted OR 2.78, 95%CI 1.23–5.12) and sisters (adjusted OR 2.03, 95%CI 1.26–3.97), but also non-lung cancer in females and mothers (OR 2.00, 95%CI 1.26–3.01, and OR 2.34, 95%CI 1.28–4.40, respectively).

**Table 4:** Risk of cancer for relatives of lung cancer probands and controls

Relatives	Lung cancer OR^b ^(95% CI)	Non-lung cancer OR^b ^(95% CI)	Any cancer OR^b ^(95% CI)
All relatives^a^	2.05(1.68–2.53)**	1.41(1.08–2.13)*	1.82(1.52–2.02)**
Female relatives	2.17(1.60–3.64)**	2.00(1.26–3.01)**	2.45(1.84–3.20)**
Male relatives	1.33(1.02–2.02)*	1.03(0.78–1.62)	1.36(1.05–1.68)*
Parents	2.90(1.97–4.32)**	1.52(1.01–2.30)*	2.16(1.62–2.87)**
Fathers	2.17 (1.21–3.86)*	1.30(0.86–2.27)	1.80(1.20–2.69)**
Mothers	2.78(1.23–5.12)**	2.34(1.28–4.40)**	3.12(2.03–4.66)**
Siblings	1.65(1.19–2.18)*	1.31(0.97–2.01)*	1.51(1.28–1.80)**
Brothers	1.21(0.79–1.80)	0.92(0.72–1.68)	1.09(0.82–1.49)
Sisters	2.03(1.26–3.97)**	1.98(1.19–3.21)*	2.13(1.58–3.00)**

* p < 0.05 ** p < 0.01

^a ^Excluded probands and spouses. ^b ^OR adjusted for family size, commune of residence, birth oder, total smoky coal exposure, pack-years of smoking, COPD history, education, age, sex and years of cooking.

## Discussion

In this study, we attempted to control for as many environmental risk factors as possible for both the probands and their relatives in order to understand better the cause of the observed family aggregation. These data demonstrate an increase risk of non-lung cancer and lung cancer specifically among first-degree relatives of lung cancer probands, and support the hypothesis that genetic factors play a role in the etiology of lung cancer. Tokuhata and Lilienfeld[2] provided the first epidemiologic evidence to support the hypothesis was provided, and reported a significantly increased risk of respiration cancer among relatives of lung cancer patients compared to age-, race-, and sex-matched controls. The risk for cancer in general, however, was not increased. The present authors[3] recently reports similar findings. Lynch et al.[5,15] reported on the familial risk of cancer in relatives of lung cancer probands and probands with other smoking-related cancers (i.e. bladder, pancreas, oral cavity). They found no increased risk for lung cancer when considered separately. However, the risk for cancers of all anatomic sites was significantly increased among the relatives of lung cancer probands (p < 0.001). This increased risk was not evident for relatives of probands with other smoking-associated cancers, nor were the cancers themselves smoking-related. The authors conclude that the data are consistent with the hypothesis of an underlying susceptibility to malignancy in these families. Unlike Lynch et al.[5], the present study again reports an increased risk for lung cancer, and also notes an increased risk for cancer at several anatomic sites in relatives of lung cancer probands. Even after excluding persons with lung cancer and controlling for the assessed environmental exposures, a statistically significant excess risk of cancer was evident.

Our findings also suggest that female relatives (especially mothers) of lung cancer cases are at higher risk for lung cancer than male relatives. Previous studies have indicated that having a first-degree relative with lung cancer was significantly associated with lung cancer among non-smokers[3,6,16-18]. Why the apparently stronger association between lung cancer and lung cancer history in female relatives are unknown. Since certain smoky coal exposure and cooking habits have been implicated as an important risk factor for lung cancer among Chinese women[19]. Genetic factors might influence the susceptibility to environmental carcingens. Excess of 10 carcinogenic chemicals were identified in cooking and smoky coal fume and these shown to interact with one another[20,21] and these possible effects need to be evaluated further.

Findings of familial aggregation and statistical evidence for a major gene [22-24] have led to the search for high-penetrant, rare, single genes for lung cancer and low-penetrant, high-frequency, susceptibility genes for lung cancer[25]. Some of the most widely and recently studied polymorphic loci coding for phase I and II enzymes involved in the activation and conjugation of carcinogen are cytochrome P4501A1 (CYP1A1), glutathione S-transferase M1 (GSTM1), myeloperoxidase (MPO), and NAD(P)H: quinone oxidoreductase (NQO1)[25]. These genes encoding enzymes that are associated with carcinogen metabolism and DNA repair have been the focus of research into possible susceptibility genes for lung cancer and othe cancer. Furthermore, the recent findings show that the homozygous GSTP1 Ile105Val genetype was significantly under-represented in NSCLC compared with controls especially in females, and neither GSTM1 nor MPO genotypes affected the overall risk of NSCLC, and the MPO and CYP1A1 risk genotypes interacted to increase overall risk of NSCLC[26]. Multiple genes of modest effect interact to confer genomic-based susceptibility to lung cancer. It has been hypothesized that differences in susceptibility to carcinogens result from an individual's ability to form genotoxic intermediates, to detoxify these intermediates, and to repair damaged DNA. Polymorphisms in genes coding for the enzymes that drive these processes are likely candidate susceptibility genes. They could not only increase the lung cancer risk but also affect the risk of other cancers. Identifying and testing specific markers using a linkage analysis to confirm the gene involved in the development and progression of lung cancer, particularly to explore the interaction of gene-gene and gene-environment, should be a high future priority.

## Abbreviations

ICD, international classification of diseases; COPD, chronic obstructive pulmonary disease; OR, odds ratio; CI, confidence interval; GST, glutathione S-transferase; CYP, cytochrome P450; MPO, myeloperoxidase and NQO1, NAD(P)H: quinone oxidoreductase

## Competing interests

The author(s) declare that they have no competing interests.

## Authors' contributions

YJ designed the study and drafted the manuscript. YX carried out the statistical analysis. MX collected the data of lung cancer cases and controls. SX Participated in the design of the study and helped to draft the manuscript. All authors read and approved the final manuscript.

## Pre-publication history

The pre-publication history for this paper can be accessed here:


